# NURSE-LED DEMENTIA MEDICAL HOME: WHAT IS IT LIKE FOR CLIENTS?

**DOI:** 10.1093/geroni/igz038.428

**Published:** 2019-11-08

**Authors:** Mariya A Kovaleva, Melinda Higgins, Bonnie M Jennings, Mi-Kyung Song, Carolyn Clevenger, Patricia C Griffiths, Ken Hepburn

**Affiliations:** 1 Vanderbilt University School of Nursing, Nashville, Tennessee, United States; 2 Nell Hodgson Woodruff School of Nursing, Emory University, Atlanta, Georgia, United States; 3 Emory University, Atlanta, Georgia, United States; 4 Emory University School of Medicine, Atlanta, Georgia, United States

## Abstract

The Integrated Memory Care Clinic (IMCC) at Emory Healthcare is a patient-centered medical home led by advanced practice registered nurses (APRNs) who provide both dementia care and primary care. In this prospective longitudinal cohort study we aimed to evaluate caregivers’ and their persons’ living with dementia (PLWD) (reported by caregivers) experiences at the IMCC. Changes in caregivers’ psychological well-being and health status and in PLWDs’ quality of life and neuropsychiatric symptoms were explored via three assessments during the clients’ first year at the IMCC. Forty-nine caregivers completed baseline assessments, including a sociodemographic questionnaire and established instruments. Mixed linear models were used to examine changes in caregiver- and PLWD-centered variables. With time as the only predictor and with full baseline sample included, significant changes were observed in caregivers’ distress regarding their PLWDs’ delusions (p=0.048) and in caregivers’ distress regarding their PLWDs’ anxiety (p=0.018). Additionally, significant changes were observed in PLWDs’ severity of delusions (p=0.032), depression (p<0.001), and total symptom severity (p=0.005). For outcomes that changed significantly over time, we explored whether time still significantly predicted changes when controlling for two variables deemed clinically important: the total number of PLWD’s chronic comorbidities besides dementia at baseline and the total number of visits the dyads made to the clinic during the study. Controlling for the total number of visits to the IMCC cancelled significant improvement in caregivers’ distress regarding PLWDs’ delusions. While most variables remained unchanged, several important symptom-related outcomes improved rapidly upon clinic enrollment, indicating potentially efficacious symptom management at the IMCC.

